# A new *Caenorhabditis elegans* model of human huntingtin 513 aggregation and toxicity in body wall muscles

**DOI:** 10.1371/journal.pone.0173644

**Published:** 2017-03-10

**Authors:** Amy L. Lee, Hailey M. Ung, L. Paul Sands, Elise A. Kikis

**Affiliations:** Biology Department, The University of the South, Sewanee, TN, United States of America; University of Pittsburgh School of Medicine, UNITED STATES

## Abstract

Expanded polyglutamine repeats in different proteins are the known determinants of at least nine progressive neurodegenerative disorders whose symptoms include cognitive and motor impairment that worsen as patients age. One such disorder is Huntington’s Disease (HD) that is caused by a polyglutamine expansion in the human huntingtin protein (htt). The polyglutamine expansion destabilizes htt leading to protein misfolding, which in turn triggers neurodegeneration and the disruption of energy metabolism in muscle cells. However, the molecular mechanisms that underlie htt proteotoxicity have been somewhat elusive, and the muscle phenotypes have not been well studied. To generate tools to elucidate the basis for muscle dysfunction, we engineered *Caenorhabditis elegans* to express a disease-associated 513 amino acid fragment of human htt in body wall muscle cells. We show that this htt fragment aggregates in *C*. *elegans* in a polyglutamine length-dependent manner and is toxic. Toxicity manifests as motor impairment and a shortened lifespan. Compared to previous models, the data suggest that the protein context in which a polyglutamine tract is embedded alters aggregation propensity and toxicity, likely by affecting interactions with the muscle cell environment.

## Introduction

George Huntington first described what subsequently became known as Huntington’s Disease (HD) in 1872 in a paper titled “On Chorea” [1]. In that landmark paper, Huntington described disease symptoms as dance-like spasmodic movements that typically manifest at around 40 years of age. He also noted that HD leaves the affected patient “but a quivering wreck of his former self” due to neurodegeneration and concomitant cognitive decline before causing premature death [1]. In 1993, the single autosomal gene that causes HD became the first human disease locus to be precisely identified. The locus was revealed via map-based cloning and the HD-associated dominant allele was shown to contain an expanded trinucleotide repeat [2]. This trinucloetide repeat encodes a destabilizing expansion of a polyglutamine (polyQ) tract in the huntingtin (htt) protein.

Examination of post-mortem brain tissue obtained from affected individuals revealed the conversion of the normally soluble htt protein monomers into insoluble inclusions of aggregated htt [3]. Similar aggregates, albeit of different proteins, are also hallmarks of other polyQ disorders including various spinal cerebellar ataxias and spinal and bulbar muscular atrophy. Furthermore, Alzheimer’s Disease, Parkinson’s Disease, Amyotrophic Lateral Sclerosis, and others are also characterized by the aggregation and toxicity of damaged or misfolded protein. The similarities in the nature of these diseases led to the coining of the general term “conformational diseases” to describe those caused in some way by protein misfolding [4].

The aggregation and associated htt proteotoxicity begins in mid to late life, when disease symptoms are first experienced. This suggests that aging is an important risk factor for disease and a likely trigger for htt protein aggregation/toxicity. The ability of cells and organisms to buffer against protein misfolding is thought to decline over time resulting in an impaired ability of older neurons to mitigate the toxic effects of the polyQ-expanded htt protein [5–7]. Considerable evidence demonstrates that the overexpression of molecular chaperones or the induction of the heat shock response can at least partially alleviate the toxicity of proteins with an expanded polyQ tract [8–15].

The polyQ-expanded form of the htt protein may trigger cell death via the initiation of an apoptotic pathway. This is evidenced by an increase in DNA fragmentation [16] and the initiation of a caspase cascade in cells expressing mutant htt [17]. In fact, the htt protein and other neurodegenerative disease-associated polyglutamine-containing proteins have been shown to undergo proteolytic cleavage by the pro-apoptotic caspases in a polyQ length-dependent manner [18]. Importantly, caspase-1 and caspase-3 both cleave the N-terminal domain of htt, with caspase-3 cleaving specifically at amino acid position 513, liberating a potentially toxic polyQ-containing fragment of that size [19]. Importantly, treatment of HEK 293 T cells expressing the polyQ-expanded htt protein with caspsase inhibitors led to a decrease in htt toxicity [20]. These findings led to the “toxic fragment hypothesis” that htt cleavage by caspases is a crucial step in HD pathology.

Do various fragments of the htt protein differ in aggregation propensity? We know more about htt exon 1 aggregation than that of other fragments. Htt exon 1 has been identified in the brains of HD mice [21] and shown to be either the result of proteolysis or of translation from an aberrantly processed mRNA [22]. It has been shown to aggregate in a two-step process *in vitro*, undergoing an initial nucleation phase of aggregation involving the formation of spherical oligomers followed by protofibril-like structures containing up to 2600 individual molecules of htt exon 1 protein [23]. Additionally, htt exon 1 formed small aggregates consisting of short fibrils in cells grown in tissue culture [24].

To study the pathogenic mechanisms underlying the toxicity of the human htt protein *in vivo*, animal models have been generated in which htt, or N-terminal fragments of htt, were expressed in mice, *Drosophila melanogaster* or *Caenorhabditis elegans*. One of the earliest and most well studied animal models is the R6/2 mouse that expresses the first ~90 amino acids of the human htt protein containing an expanded polyQ tract of 144 glutamines [25]. The R6/2 mouse recapitulates the progressive neurodegeneration observed in HD patients and has been used as a drug discovery tool [26].

Since the time that the R6/2 mouse was first published, many *C*. *elegans* models of htt aggregation and toxicity have likewise been developed. The first expressed the N-terminal 171 amino acids of htt (Htt171) in sensory neurons including the ASH, ASI, PHA, and PHB neurons under the control of the *osm-10* promoter [27]. Htt171 was shown to aggregate in and be toxic to ASH neurons in a polyQ length-dependent manner [27].

Another *C*. *elegans* htt model was developed in which only the first 57 amino acids of the htt protein (Htt57) with short (Q19) and long (Q88 and Q128) polyQ tract lengths was tagged with GFP for visualization and expressed in mechanosensory neurons under the control of the *mec-3* promoter [28]. This Htt57 fragment resulted in the dysfunction, but not death, of PLM neurons. Therefore, certain neuronal subtypes may be more susceptible to the toxic effects of polyQ-containing proteins than others and neurodegeneration may not be the only way that toxicity manifests. Additionally, aggregates predominated in neuronal processes rather than cell bodies, resulting in abnormal axonal morphologies [28].

The most recent *C*. *elegans* htt model to be described expresses htt exon 1 with Q28, Q55, or Q74 in body wall muscle cells, and is fused to GFP for visualization [29]. Until now, that was the only model to express a fragment of the htt protein in muscle cells. Similar to findings in neurons, the htt exon 1 fragment displayed polyQ length-dependent aggregation and toxicity in body wall muscle cells [29].

Two additional *C*. *elegans* models published in 2002 [30] and 2006 [31] cannot be considered htt models *per se* because they do not contain htt protein sequence. Instead, they express different polyQ tract-lengths fused to GFP for visualization (referred to herein as polyQ alone). As such, they are generic models, modeling polyQ toxicity irrespective of the normal protein context. PolyQ alone was shown to aggregate in body wall muscle cells [30] or neurons [31] in a polyQ length-dependent manner and to cause cellular dysfunction in both tissues.

For the sake of comparison, a complete list of previously published *C*. *elegans* models of polyQ alone or polyQ in the context of human htt is shown (**Table 1**). Each model differs with respect to htt fragment length, the presence of a fluorescent tag, and the cell type in which htt is expressed.

**Table 1:** Previously published Htt or polyQ alone *C*. *elegans* models.

Expressed Protein	Tissue/Cell Type	PolyQ lengths	Phenotypes	References
Htt171	Sensory neurons (ASH, ASI, PHA, PHB)	Q2, Q23, Q95, Q150	PolyQ length-dependent aggregation and toxicity. Age-dependent aggregation of Q150 in ASH neurons.	Faber, et. al., *Proc*. *Natl*. *Acad*. *Sci*., 1999
Htt57-YFP	Touch Receptor Neurons (AVM, ALML, ALMR, PVM, PLML, PLMR)	Q19, Q88, Q128	PolyQ length-dependent aggregation and toxicity in PLM tail mechanosensory neurons	Parker, et. al., *Proc*. *Natl*. *Acad*. *Sci*., 2001
polyQ-YFP	Body wall muscle cells	Q19, Q29, Q33, Q35, Q40, Q44, Q64, Q82	PolyQ length-dependent and age-dependent aggregation and toxicity.	Morley, etl. al., *Proc*. *Natl*. *Acad*. *Sci*., 2002
polyQ-YFP	All Neurons	Q19, Q35, Q40, Q67, Q86	PolyQ length-dependent aggregation and toxicity. No age-dependent changes observed.	Brignull, et. al., J Neurosci., 2006
GFP-Htt exon 1	Body wall muscle cells	Q28, Q55, Q74	PolyQ length-dependent aggregation and toxicity.	Wang, et. al., *Hum*. *Mol*. *Genet*., 2006

Each of the *C*. *elegans* models described here has contributed in its own way to our understanding of the mechanisms underlying polyQ toxicity and the genetic and physical interactions between polyQ-containing proteins and the cellular environment. For example, aggregation of polyQ alone responds to changes in the overall load of misfolded protein [32, 33] and decreases when molecular chaperones are upregulated [9, 34] or when neuronal signaling is suppressed [12]. But do these findings with polyQ alone translate to other htt models including vertebrates?

To address this, a genome-wide RNAi screen was performed to identify gene inactivations that led to either an increase or decrease in Htt57-GFP toxicity as measured by PLM neuron function [35]. That screen revealed 49 genes that also suppress htt toxicity in mice, providing some of the first evidence that findings in *C*. *elegans* can directly translate to mammalian models. Likewise, important conserved signaling pathways, such as that associated with β-catenin, suppressed Htt57-GFP toxicity in PLM neurons via interaction with the forkhead transcription factor FOXO/DAF-16 [36]. In an elegant study published recently, Tourette *et*. *al*. showed that the Wnt receptor is upregulated in animals expressing Htt57-GFP in mechanosensory neurons, thereby leading to the observed decrease in FOXO activity [37]. As in previous studies, these findings in *C*. *elegans* nicely translated to mouse models [37].

Most of what we know about HD relates to neurodegeneration. However, the htt protein is ubiquitously expressed and recent studies point to early pathological hallmarks of disease being caused by previously overlooked changes in metabolic pathways in muscle cells [38]. Specifically, changes to mitochondrial function in muscle cells were observed in pre-symptomatic gene positive individuals [39]. Likewise, ATP/phosphocreatine levels were decreased in the muscles of both pre- and post-symptomatic HD patients [40]. Together, these metabolic deficiencies are thought to contribute to muscle wasting in patients and HD mice.

Typically, the mouse has been the model system of choice to study muscle phenotypes associated with HD. This is partly because few suitable invertebrate models existed. Only one of the *C*. *elegans* models described above expresses a htt fragment in muscle cells [29]. That model was used to show that the RNAi-mediated knockdown of the mitochondrial fission gene Drp-1 rescued the muscle dysfunction that was caused by htt exon 1 [41].

Htt exon 1 encodes an N-terminal 17 amino acid region, a polyQ repeat, and a poly proline rich domain (PRD). Longer fragments including the caspase cleavage fragments Htt513 and Htt586 have these same features but also have a series of structurally ordered segments containing HEAT repeats separated by intrinsically disordered regions that include the sites of proteolysis [42]. HEAT repeats are present in a variety of proteins and are known to participate in protein-protein interactions. Nonetheless, the exact number of HEAT repeats, or their binding partners, is not known for the htt protein [22]. Despite their hypothesized role in HD pathology, caspase cleavage products of htt have never been directly examined for toxicity in *C*. *elegans* or any other model system. That being said, the full length htt protein was expressed in mice [43]. Because of the potential importance of the htt HEAT repeats in mediating interactions with other cellular factors, having an invertebrate model expressing a long htt fragment should allow for a more comprehensive view of htt action.

To that end, we describe here a new *C*. *elegans* model for htt aggregation and toxicity, in which the naturally occurring 513 amino acid caspase cleavage product, herein referred to as Htt513, was engineered to have either wild type or expanded polyQ tracts and to be expressed in body wall muscle cells. We show that aggregation of the Htt513 fragment in body wall muscle cells is a polyQ length-dependent process. We also demonstrate that aggregation is associated with motor defects and a shortened lifespan, indicating that Htt513 proteotoxicity is likewise polyQ length-dependent. This model represents a new tool to study the molecular mechanisms of htt-associated muscle dysfunction as caused by a long and biologically relevant htt fragment.

## Materials and methods

### Plasmid constructs

The P_*unc-54*_Htt513(Q_n_)::YFP gene constructs were generated by PCR amplification of the Htt513 fragment from a previously published full length cDNA clone of the human htt gene [44]. The primers used for PCR amplification were 5’ AATACCGCGGATGGCGACCCTGGAAAAGCTG, which contains a 5’ SacII site and 5’ TTATACCGGTCCATCCACTGAGTCCGCCTGCAG, which contains a 3’ AgeI site. The resultant PCR products were cloned into the corresponding restriction sites of the previously described pPD30.38Q0::YFP plasmid which is a *C*. *elegans* vector containing the *unc-54* promoter driving YFP expression in body wall muscle cells [30].

The resulting sequence of the htt protein fragment used in this study was: MATLEKLMKAFESLKSFQ(n)PPPPPPPPPPPQLPQPPPQAQPLLPQPQPPPPPPPPPPGPAVAEEPLHRPKKELSATKKDRVNHCLTICENIVAQSVRNSPEFQKLLGIAMELFLLCSDDAESDVRMVADECLNKVIKALMDSNLPRLQLELYKEIKKNGAPRSLRAALWRFAELAHLVRPQKCRPYLVNLLPCLTRTSKRPEESVQETLAAAVPKIMASFGNFANDNEIKVLLKAFIANLKSSSPTIRRTAAGSAVSICQHSRRTQYFYSWLLNVLLGLLVPVEDEHSTLLILGVLLTLRYLVPLLQQQVKDTSLKGSFGVTRKEMEVSPSAEQLVQVYELTLHHTQHQDHNVVTGALELLQQLFRTPPPELLQTLTAVGGIGQLTAAKEESGGRSRSGSIVELIAGGGSSCSPVLSRKQKGKVLLGEEEALEDDSESRSDVSSSALTASVKDEISGELAASSGVSTPGSAGHDIITEQPRSQHTLQADSVD (Uniprot ID P42858).

Plasmid constructs were generated that had 15 or 128 CAG repeats, indicated as P_*unc-54*_Htt513(Q_15_)::YFP or P_*unc-54*_Htt513(Q_128_)::YFP, respectively. The expressed proteins are herein referred to as Htt513(Q_15_) or Htt513(Q_128_) for simplicity.

### *C*. *elegans* strains, crosses, and culture

*C*. *elegans* were cultured according the standard methods [45]. In short, animals were maintained at 20°C or 15°C on NGM agar plates seeded with *E*. *coli* (OP50) obtained from the *Caenorhabditis* Genetics Center (St. Paul, Minnesota). The wild type strain used was the Bristol N2 isolate. The P_*unc-54*_YFP line (herein referred to as YFP) was previously published and expresses an integrated YFP transgene in body wall muscle cells [30]. To generate transgenic animals, 50ng/μL of DNA encoding P_*unc-54*_Htt513(Q_15_)::YFP or P_*unc-54*_Htt513(Q_128_)::YFP were microinjected into the gonads of adult wild type hermaphrodites to generate multiple (at least 5) independent lines transmitting extrachromosomal arrays. Integrated P_*unc-54*_Htt513(Q15)::YFP (EAK102) or P_*unc-54*_Htt513(Q128)::YFP (EAK103) lines were generated by gamma irradiation followed by backcrossing to N2 animals for at least three generations to ensure a wild type genetic background free of secondary mutations.

### Fluorescence microscopy

To obtain confocal z-stacks of transgenic *C*. *elegans*, animals were fixed with 4% paraformaldehyde and actin filaments were stained with phalloidin (Molecular Probes/Life Technologies, Grand Island, NY) as previously described [7]. Imaging was with a Leica SP8 confocal microscope (Wetzlar, Germany) using a 40X oil immersion objective. For Fluorescence Recovery after Photobleaching (FRAP) of Htt513(Q_128_) foci in living animals, day 1 adults were immobilized with 2mM levamisole, mounted on 2% agarose pads, and covered with a coverslip. FRAP was performed on a Leica SP8 confocal microscope with a 63X oil immersion objective (Wetzlar, Germany). Data were analyzed as previously described [31]. For analysis of aggregation over time, images were obtained with a Ziess Axio Observer A1 (Oberkochen, Germany) inverted compound fluorescence microscope using a 20X objective. Micrographs of small regions of the animal were stitched together manually to obtain images of whole animals.

### Quantification of aggregate number

Quantification of aggregate number was performed on live animals at day 1 of adulthood. Fluorescent micrographs were obtained with a Ziess Axio Observer (Oberkochen, Germany) as described above and aggregate number in each image was determined by counting the fluorescent foci. A minimum n-number of n = 50 animals was examined for each genotype.

### Motility assays

Our measure of motility was thrashing rate in liquid. Individual animals harboring extrachromsomal or integrated arrays were examined at days 1, 4 and 8 of adulthood. They were picked from petri plates to a 10μL drop of M9 on a microscope slide. Animals were given 30s to recover from passaging before counting thrashes. A thrash was scored each time that the head crossed the vertical midline of the body. The total number of thrashes was counted for 60s. An n-number of n = 30–50 animals was assayed for each genotype. Statistical analyses were performed using R-studio.

### Lifespan assays

Animals were acclimated at 20°C for at least two generations before initiating lifespan analyses. Forty age-matched L4 larvae were placed on NGM plates seeded with *E*. *coli* (OP50) and cultured at 20°C. During the reproductive time of their life cycle, animals were transferred away from their progeny by daily passaging to freshly seeded plates. Post-reproductive adults were passaged as needed to prevent starvation. Animals were scored as deceased when they no longer moved upon prodding the head several times with a platinum wire as described previously [46]. Animals that crawled off the plate were censored in statistical analyses by adjusting the total population to the number of animals seen on the plate on a given day. Data were analyzed and plotted using R-studio.

### Western blot analysis

100 fluorescent L4 larval stage animals were frozen at -80°C in M9 overnight and thawed on ice before boiling in Laemmli sample buffer for 5min. Samples were centrifuged at 10,000Xg for 5min prior to loading on 10% polyacrylamide gels (SDS-PAGE). Following transfer to PVDF filters, immunodetection was with an IRDye800 conjugated anti-GFP antibody (cat# 600-432-215) from Rockland Immunochemicals, Inc. (Gilbertsville, PA) or with the anti-polyQ antibody 3B5H10 from Sigma (St. Louis, MO). Visualization was with an Odyssey system from Li-Cor (Lincoln, NE). Three biological replicates were performed and average band intensity for the three replicates was determined using the Odyssey system.

### mRNA analysis

Ten day 1 and day 4 adult animals were frozen in liquid nitrogen and stored at -80°C in M9 before RNA isolation using Trizol^TM^ Reagent. RNA was treated with DNase I using the DNA-*free*^TM^ DNA removal kit from Thermo Fisher Scientific, Inc. (Waltham, MA) according to the manufacturers instructions. cDNA was synthesized using iScript Reverse Transcriptase (Bio-Rad Laboratories, Hercules, CA) according to the manufacturers instructions. The Bio-Rad cyber green master mix and the Bio-Rad MyIQ cycler were used for quantitative PCR (qPCR) of *yfp* or actin as a loading control. The YFP primers were 5’ ATGGTGAGCAAGGGCGAGGAGCTGTTC and 5’ GGTGGCATCGCCCTCGCCCTCGCCG.

The actin primers were 5’ ATCACCGCTCTTGCCCCATC and 5’ GGCCGGACTCGTCGTATTCTT. Three independent biological replicates were performed (each in technical triplicate) and the data averaged.

## Results and discussion

### Expression of Htt513 in *C*. *elegans* body wall muscle cells

**Fig 1:**
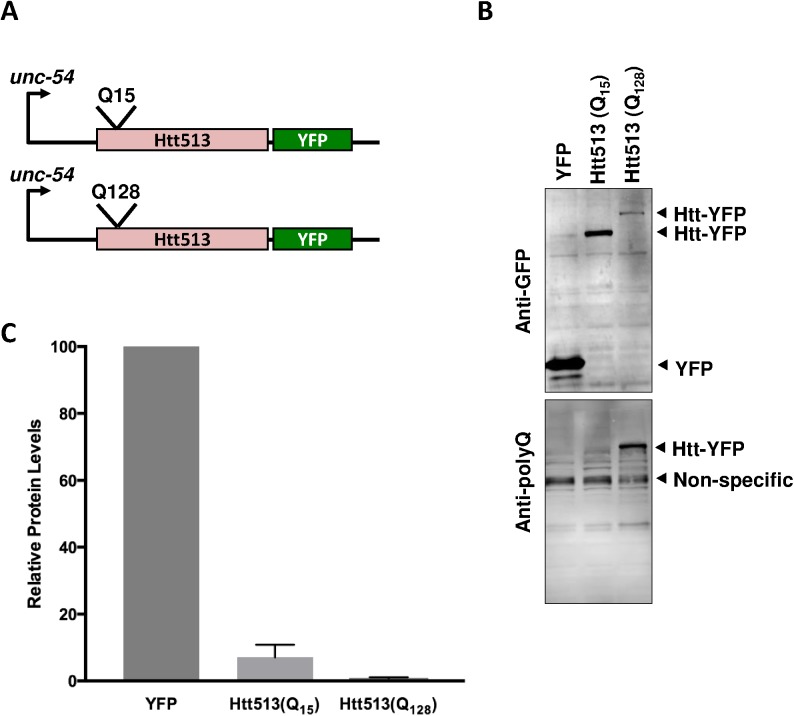
Htt513 protein expression in *C*. *elegans* body wall muscle cells. (A) Schematic representation of gene constructs for Htt513(Q_n_) expression in *C*. *elegans* body wall muscle cells. The polyQ-containing N-terminal domain including the first 513 amino acids of human htt was translationally fused to YFP and expressed in body wall muscle cells under the control of the *unc-54* promoter. Short (Q_15_) and long (Q_128_) polyQ tracts were generated. (B) A representative immunoblot was probed with an anti-GFP antibody (top) and reprobed with an anti-expanded polyQ antibody (bottom). (C) Quantification of protein bands detected with the anti-GFP antibody. Data represent means of three biological replicates (n = 3) and error bars represent standard error of the mean.

To express the first 513 amino acids of human htt (Htt513) in *C*. *elegans* body wall muscle cells, we utilized the myosin heavy chain promoter, *unc-54*. YFP was translationally fused to the C-terminus of Htt513 allowing us to visualize the protein in live animals. Because polyQ length is known to affect aggregation and toxicity [47–49], we generated two different polyQ tract lengths for the purpose of comparison. One was short (Q_15_) and in a range not usually associated with HD. The other was much longer (Q_128_) and in the range of pathogenesis (**Fig 1A**). Formally, the resultant proteins are Htt513(Q_15_)::YFP and Htt513(Q_128_)::YFP, respectively. However, for simplicity’s sake we refer to them here as Htt513(Q_15_) and Htt513(Q_128_).

Because protein aggregation is a concentration-dependent process, we wanted to ensure that our two Htt513 proteins were expressed at similar levels. We thus examined their relative levels in integrated transgenic lines by immunoblot analysis with an anti-GFP antibody that reliably cross-reacts with YFP. Both Htt513(Q_15_) and Htt513(Q_128_) accumulated to substantially lower levels than YFP alone (**Fig 1B**). Specifically, Htt513(Q_15_) accumulated an average of 7% of YFP levels and Htt513(Q_128_) accumulated an average of only 0.8% of YFP levels (**Fig 1C**). In fact, Htt513(Q_128_) protein levels were so low it was detectable only as a very faint band in an immunoblot analysis compared to the readily detected Htt513(Q_15_) protein. Because Htt513(Q_128_) was so difficult to detect, we re-probed the immunoblot with a very sensitive antibody that was raised against expanded polyQ tracts. As expected, the anti-expanded polyQ antibody recognized Htt513(Q_128_), but not Htt513(Q_15_), demonstrating that the faint Htt513(Q_128_) band on the anti-GFP immunoblot was indeed Htt513(Q_128_) (**Fig 1B**).

**Fig 2:**
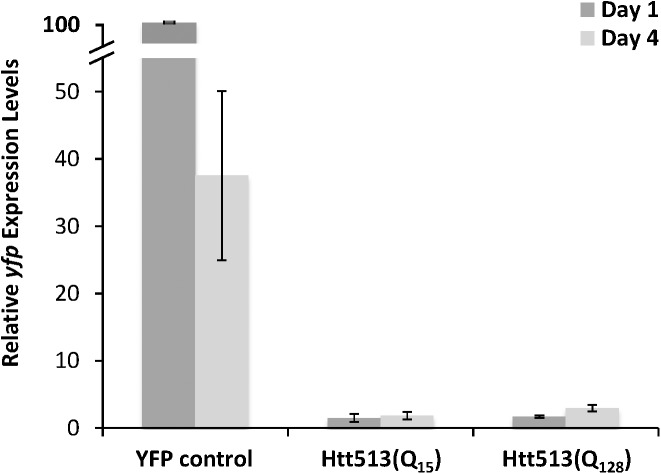
Levels of Htt513 mRNA are low in adult animals. Total RNA was isolated from equivalent numbers of YFP control animals and animals expressing Htt513(Q_15_) or Htt513(Q_128_) at day 1 or day 4 of adulthood. qRT-PCR was performed with primers for YFP and expression levels were normalized to actin and plotted relative to the YFP control at day 1 of adulthood, which was set equal to 100%. The data represent means of three biological replicates and error bars represent standard error of the mean.

To confirm that the steady-state protein levels for Htt513(Q_15_) and Htt(Q_128_) were not affected by the positions at which the transgenes inserted, we also examined protein levels in lines transmitting non-integrated extrachromosomal arrays. The steady-state levels of Htt513(Q_15_) and Htt513(Q_128_) protein were examined in at least 5 independent extrachromosomal array lines and no overt differences were observed between lines harboring the same transgene. Specifically, the levels of Htt513(Q_15_) were much lower than YFP alone, similar to the results obtained from integrated lines. Importantly, Htt513(Q_128_) was only detected with the anti-expanded polyQ antibody and not with the anti-GFP antibody as shown in a representative immunoblot (**S1 Fig**). At face value, this lack of detection with the anti-GFP antibody indicates extremely low Htt513(Q_128_) protein levels, although an inhibitory effect of the expanded polyQ tract on the binding of the anti-GFP antibody cannot be ruled out. Therefore, we also examined mRNA levels using qRT-PCR. Both Htt513(Q_15_) and Htt513(Q_128_) mRNA levels accumulated to only ~2% of control levels at day 1 and day 4 of adulthood (**Fig 2**). Because Htt513(Q_15_) and Htt513(Q_128_) were expressed at similar levels, we can compare these lines with respect to aggregation and toxicity.

### Htt513 aggregates in a polyglutamine length-dependent manner in *C*. *elegans* body wall muscle cells

**Fig 3:**
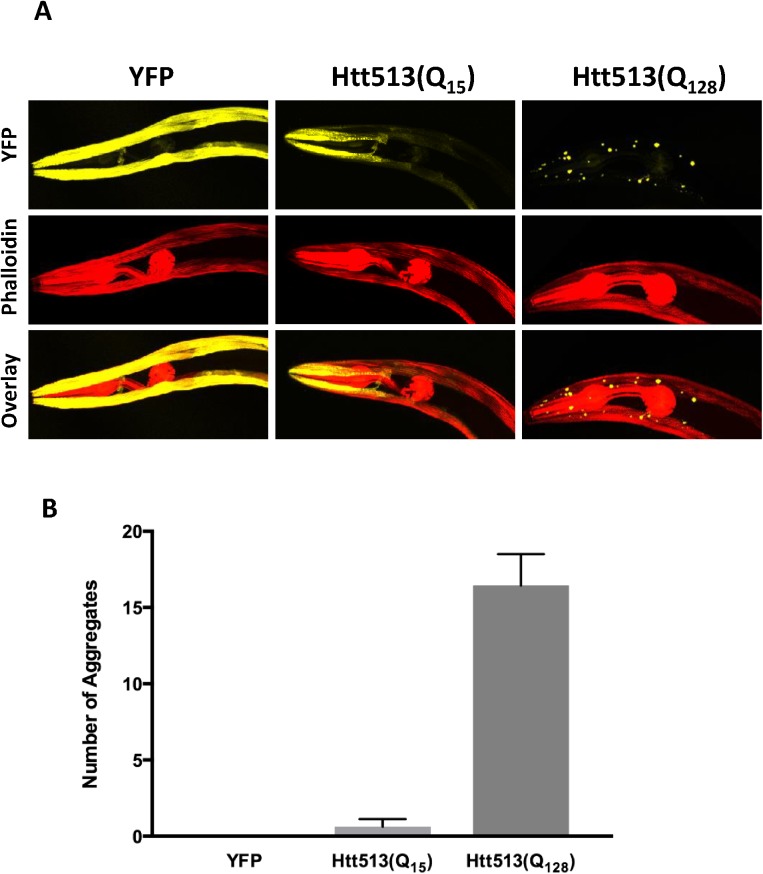
The Htt513 protein forms large visible foci in body wall muscle cells in a polyglutamine length-dependent manner. (A) Z-stack projections. Day 1 adult animals were fixed, stained with phalloidin, and z-stacks of head regions were imaged with a confocal microscope. YFP is shown in yellow and phalloidin-stained actin filaments are shown in red. (B) Quantification of fluorescent foci number in whole animals expressing Htt513(Q_15_) or Htt513(Q_128_). The data represent average foci number across 50 individuals (n = 50). Error bars represent standard error of the mean.

For our model to useful, it should recapitulate some of the key features of HD, such as htt aggregation. In patients, a threshold length of 35 glutamines has been described such that longer polyQ tracts result in htt aggregation. Thus, if the aggregation dynamics for Htt513 in *C*. *elegans* are similar to that observed in patients, we would not expect Htt513(Q_15_) to aggregate in *C*. *elegans* body wall muscle cells, but we would expect Htt513(Q_128_) to aggregate. Consistent with this, Htt513(Q_15_) yielded primarily diffuse fluorescence at day 1 of adulthood, whereas Htt513(Q_128_) formed fluorescent foci (**Fig 3A**). Upon counting the number of foci, we found that the Htt513(Q_15_) integrated line had an average of less than one per animal compared to the Htt513(Q_128_) integrated line that had an average of 26 (**Fig 2B**). These counts were similar to those of corresponding lines harboring extrachromosomal arrays (**S2 Fig**). Such foci are suggestive of Htt513(Q_128_) aggregation.

**Fig 4:**
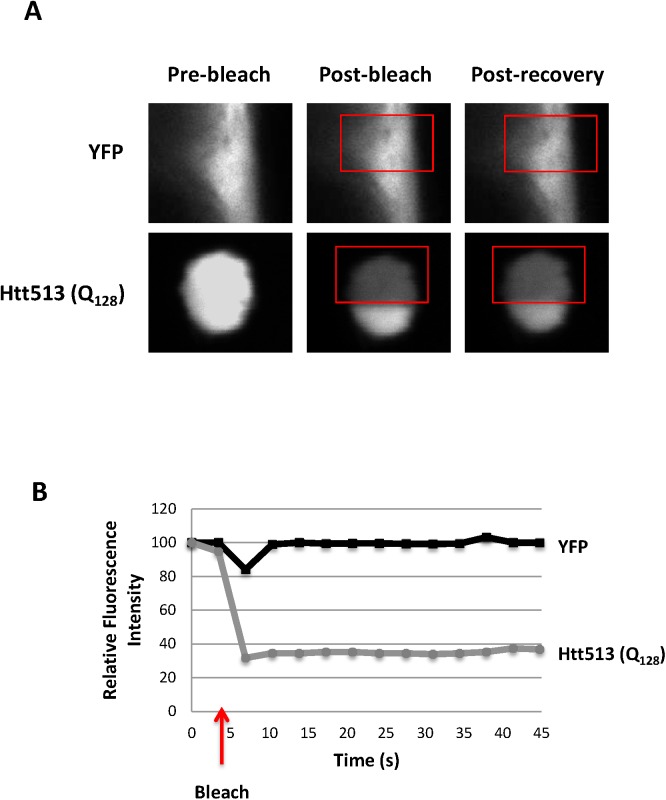
FRAP reveals Htt513(Q_128_) aggregates in *C*. *elegans* body wall muscle cells. Fluorescence Recovery After Photobleaching (FRAP) was performed on regions of diffuse fluorescence in animals expressing YFP alone or on individual fluorescent foci in Htt513(Q_128_) animals. (A) Representative images before bleaching (pre-bleach), immediately following bleaching (post-bleach) and after a 45s recovery (post-recovery). Red boxes indicate the regions subjected to photobleaching. (B) Quantification of relative fluorescence intensity over a 45s FRAP time course. Data represent averages of at least 10 fluorescent foci (or regions of diffuse fluorescence) in different animals. Error bars represent standard error of the mean. The time of bleaching is indicated with an arrow.

To determine if Htt513(Q_128_) formed *bona fide* protein aggregates, we performed Fluorescence Recovery After Photobleaching (FRAP) of the large, visible, Htt513(Q_128_) foci and compared their recovery time to those of areas of diffuse fluorescence in animals expressing YFP alone in body wall muscle cells. As expected for immobile protein aggregates, the Htt513(Q_128_) foci showed no recovery of fluorescence within the 45s time course that we recorded (**Fig 4**). This is in contrast to the Htt513(Q_15_) protein which did not form foci and whose diffuse fluorescence recovered rapidly after photobleaching (**S3 Fig**). Together, these data indicate that aggregation is dependent on polyQ tract length and not on other inherent characteristics of the Htt513 protein fragment.

**Fig 5:**
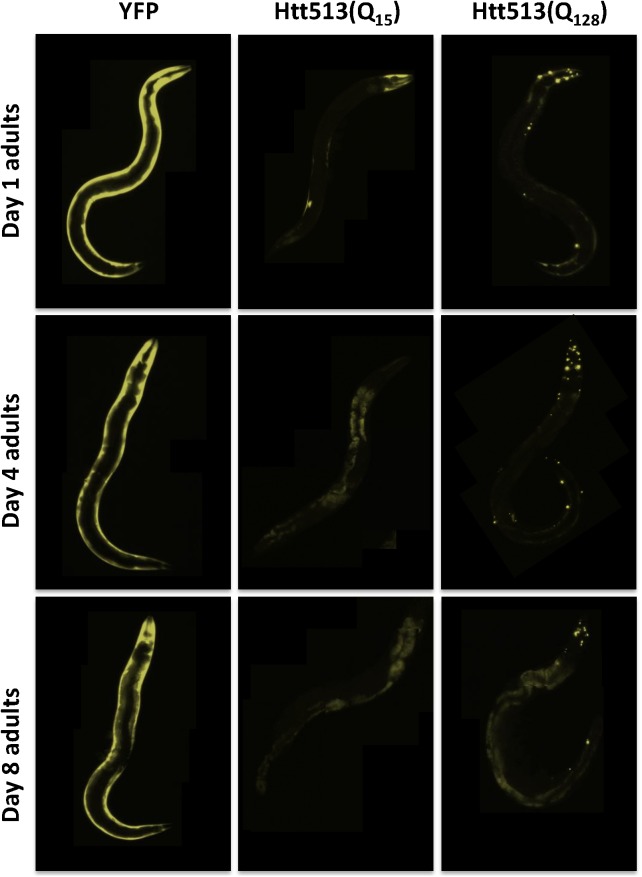
Age-dependent decline in Htt513(Q_15_) steady-state protein levels. Representative fluorescent micrographs showing YFP in live animals examined over a time course spanning days 1–8 of adulthood. Htt513(Q_15_), and Htt513(Q_128_) integrated lines were imaged with the same exposure time (300ms), while YFP alone was imaged with an exposure time 100X shorter (3ms) due to its much higher expression levels. Intestinal autoflourescence began to appear between days 4–8 of adulthood and can be seen in the animals imaged with longer exposure times.

In HD patients, aggregation is age-dependent and correlates with the onset of symptoms. To determine whether the aggregation of Htt513(Q_15_) or Htt513(Q_128_) changes during aging, we examined animals grown to days 1, 4, or 8 of adulthood. We found that Htt(Q_128_) was already aggregated at day 1 as described above (**Fig 4**), and that those aggregates persisted unchanged to day 8. Furthermore, Htt513(Q_15_) was not aggregated at day 1 of adulthood, and no age-dependent increase in aggregation was observed (**Fig 5**).

Surprisingly, instead of aggregation worsening over time, the levels of Htt513(Q_15_) protein declined to almost undetectable levels by day 4 of adulthood (**Fig 5**). A parallel drop in mRNA levels did not seem to cause this drop protein levels. qRT-PCR revealed that YFP control animals experienced a 60% decline in *yfp* mRNA between days 1 and 4 of adulthood. In contrast, both Htt513(Q_15_) and Htt513(Q_128_) transgenes expressed only 2% of control levels at day 1 of adulthood but expression did not decline during aging (**Fig 2**). In other words, Htt513(Q_15_) and Htt513(Q_128_) mRNA levels were low but steady in adult animals.

The finding that Htt513(Q_15_) protein levels decline over time despite constant mRNA levels suggests that Htt513(Q_15_) is a likely target for protein turnover. Additionally, it seems to be a better substrate for turnover than Htt513(Q_128_) or YFP alone, as both of those proteins persisted well into adulthood. This finding regarding protein stability is consistent with a recent study of the half-lives of htt protein fragments in human cells in tissue culture where extremely short N-terminal htt fragments were very stable, with half lives in the order of 16 hours, compared to a longer fragment including the first 508 amino acids of human htt that had a half life of only 1.5hrs [50]. The 513 amino acid fragment analyzed here is even longer than that, with perhaps an even shorter half-life. Furthermore, the apparently greater stability of Htt513(Q_128_) compared to Htt513(Q_15_) is likely due to increased aggregation of the polyQ-expanded protein interfering with the degradation machinery.

### Htt513(Q_128_) is toxic to *C*. *elegans* body wall muscle cells.

**Fig 6:**
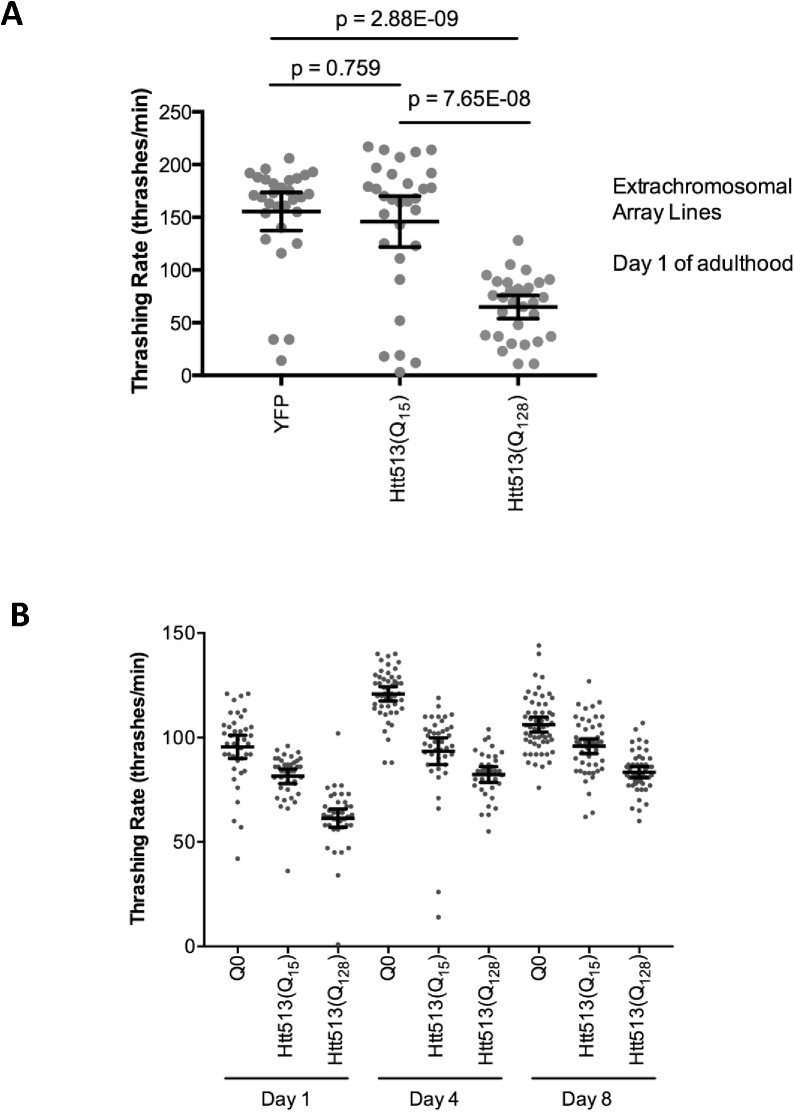
Htt513(Q_128_) is toxic to body wall muscle cells. Toxicity was determined as a function of the rate at which animals thrash in liquid. Individual data points are indicated for each genotype and age to illustrate the full range of data. Mean thrashing rate is indicated with horizontal lines. Error bars represent 95% confidence intervals. A) Thrashing rate at day 1 of adulthood for animals harboring extrachromosomal arrays. The indicated p-values are the results of Sheffé multiple comparisons post-hoc tests. ANOVA p-value = 1.23E-10. n = 30. B) Thrashing rate at days 1, 4, and 8 of adulthood for animals harboring integrated transgenes. ANOVA p-value = 1.1e-16. n = 50

Htt exon 1 was previously shown to be toxic to *C*. *elegans* body wall muscle cells, especially when it had an expanded polyQ repeat [29]. Likewise, expanded polyQ alone was also toxic to muscle cells [30]. Interestingly, aging was shown to exacerbate the toxicity of some polyQ proteins [30] but not others [51]. To determine whether Htt513 is toxic and whether toxicity is modulated by age or polyQ length, we assayed muscle cell function by measuring thrashing rate in liquid at days 1, 4, and 8 of adulthood (**Fig 6B**).

Qualitatively, Htt513(Q_15_) and Htt513(Q_128_) appeared to have slower thrashing rates than that of control animals expressing YFP alone at all time points tested. ANOVA analysis of all nine treatments yielded a p-value of 1.1e-16, so we performed post-hoc analyses in the form of *a priori* planned comparisons using the Scheffé multiple comparisons test (**Table 2**).

**Table 2:** Scheffé Multiple Comparisons Post-Hoc Test of Significance.

Treatment pairs	p-value	Significance
day 1 YFP vs. Htt513(Q_15_)	0.99	-
day 1 YFP vs. Htt513(Q_128_)	1.95E-11	***
day 4 YFP vs. Htt513(Q_15_)	2.22E-16	***
day 4 YFP vs. Htt513(Q_128_)	1.11E-16	***
day 8 YFP vs. Htt513(Q_15_)	0.001	***
day 8 YFP vs. Htt513(Q_128_)	2.28E-13	***
Htt513(Q_15_) day 1 vs. day 4	0.03	*
Htt513(Q_15_) day 1 vs. day 8	2.99E-04	***
Htt513(Q_15_) day 4 vs. day 8	9.99E-01	-
Htt513(Q_128_) day 1 vs. day 4	3.48E-08	***
Htt513(Q_128_) day 1 vs. day 8	1.95E-11	***
Htt513(Q_128_) day 4 vs. day 8	0.99	-

- p>0.05.

* p<0.05.

*** p≤0.001.

Using a p-value cut-off of less than 0.001, these data indicate that only Htt513(Q_128_) resulted in motility defects relative to YFP alone at all time points tested. In contrast, Htt513(Q_15_) appeared to be greatly affected in motility only at day 4 of adulthood, only somewhat affected at day 8, and showed no age-dependent increase in toxicity as determined by comparing the thrashing rate of Htt513(Q_15_) animals over time (**Fig 6B, Table 2**). Likewise, Htt513(Q_128_) did not increase in toxicity during aging. Instead, there was a slight motility improvement observed between days 1–4. Despite the statistically significant motility defects observed for Htt513(Q_128_) animals, no overt morphological abnormalities were detected in muscle cells via phalloidin staining of actin filaments (**Fig 3A**).

To ensure that the observed toxicity was not an artifact of the Htt513(Q_128_) transgene insertion site, we also examined the thrashing rate of animals expressing Htt513(Q_15_) and Htt513(Q_128_) from extrachromosomal arrays. We found a similar motility impairment at day 1 of adulthood (**Fig 6A**), indicating that the observed motility defect is most likely due to the toxic effects of the Htt513(Q_128_) protein.

### Animals expressing Htt513(Q_128_) have a shortened life span

**Fig 7:**
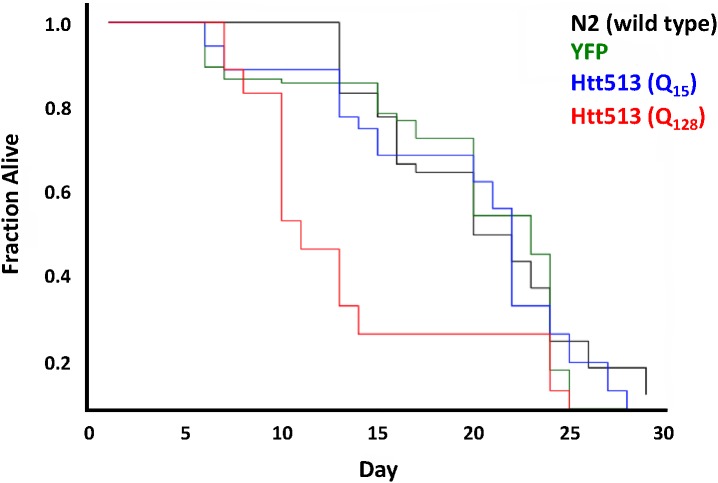
Htt513 (Q_128_) animals have a shortened lifespan. Lifespan assays were performed on populations of at least 40 individuals expressing either Htt513(Q_15_), Htt513(Q_128_), or YFP in body wall muscle as compared to wild type animals. The fraction of animals still alive at any given day is indicated.

As a complementary approach to measure the toxic effects of the Htt513(Q_128_) protein, we asked whether expression in *C*. *elegans* body wall muscle cells had any effect on lifespan. To address this, we performed a lifespan analysis of N2 (wild type), YFP, Htt513(Q_15_), and Htt513(Q_128_) animals. We observed a marked reduction in the mean lifespan of animals expressing Htt513(Q_128_) (**Fig 7**). Specifically, Htt513(Q_128_) animals had a mean lifespan of 12 days and a maximum lifespan of 25 days compared to N2, YFP, and Htt513(Q_15_) animals which all had mean lifespans of 21 days and maximum lifespan of 29, 25, and 28 days respectively. These data indicate that the toxicity of the Htt513(Q_128_) protein manifests not only as impaired muscle function but also early death. This is in contrast to the previously described C-terminal fragment of the human ataxin-3 protein which, even in its polyQ-expanded form, did not affect *C*. *elegans* lifespan despite a significant impairment of muscle function [51].

## Conclusions

We have described a new model for the aggregation and toxicity of a disease-associated fragment of the polyQ-containing htt protein in *C*. *elegans* body wall muscle cells. This is the first time that a htt fragment of this length (Htt513) was expressed in *C*. *elegans*–all other fragments have been no longer than 171 amino acids. Furthermore, this is only the second time that any htt fragment has been expressed in *C*. *elegans* body wall muscle cells. All polyQ-containing proteins expressed to date in *C*. *elegans* body wall muscle cells (polyQ alone, AT3CT, Htt exon1, and now Htt513) aggregate in a polyQ length-dependent manner and display polyQ length-dependent toxicity. One notable difference is that steady-state Htt513 protein levels seem to be lower than those of other polyQ-containing proteins in this same tissue. It is possible that Htt513 is a better substrate for protein turnover, especially in its wild type rather than polyQ-expanded form. Another notable difference is that only Htt513(Q_128_) was reported to shorten lifespan. Taken together, the data presented here suggest that the Htt513 fragment may have unique physical interactions with the cellular environment, including, but not limited to, the protein degradation machinery. As such, screens for genetically or physically interacting proteins will likely tell us a great deal about how Htt513 interfaces with the proteostasis network for which many members including molecular chaperones and neuronal signaling components have recently been identified in *C*. *elegans* [5]. Finally, this should be a useful model to study the muscle-specific effects of mutant htt.

## Supporting information

S1 Fig Expression of Htt513(Q_n_) proteins in *C. elegans* body wall muscle cells from extrachromosomal arrays. Top: Representative immunoblot probed with an anti-expanded polyQ antibody. Bottom: The same immunoblot as above probed with an anti-GFP antibody. The YFP control was expressed from an integrated transgene while Htt513(Q_15_) and Htt513(Q_128_) were expressed from extrachromosomal arrays.
(TIF)

S2 Fig Htt513 aggregation in extrachromosomal array lines. Day 1 adult animals were fixed and imaged with a compound fluorescence microscope. YFP fluorescence is shown for animals expressing Htt513(Q_15_) or Htt513(Q_128_).
(TIF)

S3 Fig Fluorescence Recovery After Photobleaching (FRAP) reveals that Htt513 (Q_15_) is not aggregated. FRAP was performed on regions of diffuse fluorescence in animals expressing YFP alone, Q19-YFP or Htt513(Q_15_). Quantification of relative fluorescence intensity over a 60s FRAP time course is shown. Data represent averages of at least 10 regions of diffuse fluorescence in different animals. Error bars represent standard error of the mean. The time of bleaching is indicated with an arrow.
(TIF)

## References

[1] Huntington G. On chorea. George Huntington, M.D. The Journal of neuropsychiatry and clinical neurosciences. 2003;15(1):109–12. Epub 2003/01/31. 10.1176/jnp.15.1.109 12556582

[2] MacDonald ME, Ambrose CM, Duyao MP, Myers RH, Lin C, Lakshmi S, et al A novel gene containing a trinucleotide repeat that is expanded and unstable on Huntington's disease chromosomes. The Huntington's Disease Collaborative Research Group. Cell. 1993;72(6):971–83. Epub 1993/03/26. 8458085 10.1016/0092-8674(93)90585-e

[3] Becher MW, Kotzuk JA, Sharp AH, Davies SW, Bates GP, Price DL, et al Intranuclear neuronal inclusions in Huntington's disease and dentatorubral and pallidoluysian atrophy: correlation between the density of inclusions and IT15 CAG triplet repeat length. Neurobiology of disease. 1998;4(6):387–97. Epub 1998/07/17. 10.1006/nbdi.1998.0168 9666478

[4] Carrell RW, Lomas DA. Conformational disease. Lancet. 1997;350(9071):134–8. Epub 1997/07/12. 10.1016/S0140-6736(97)02073-4 9228977

[5] Kikis EA. The struggle by Caenorhabditis elegans to maintain proteostasis during aging and disease. Biology direct. 2016;11(1):58 Epub 2016/11/05. PubMed Central PMCID: PMC5093949. 10.1186/s13062-016-0161-2 27809888 PMC5093949

[6] Gidalevitz T, Kikis EA, Morimoto RI. A cellular perspective on conformational disease: the role of genetic background and proteostasis networks. Current opinion in structural biology. 2010;20(1):23–32. Epub 2010/01/08. PubMed Central PMCID: PMC3050498. 10.1016/j.sbi.2009.11.001 20053547 PMC3050498

[7] Ben-Zvi A, Miller EA, Morimoto RI. Collapse of proteostasis represents an early molecular event in Caenorhabditis elegans aging. Proceedings of the National Academy of Sciences of the United States of America. 2009;106(35):14914–9. Epub 2009/08/27. PubMed Central PMCID: PMC2736453. 10.1073/pnas.0902882106 19706382 PMC2736453

[8] Bailey CK, Andriola IF, Kampinga HH, Merry DE. Molecular chaperones enhance the degradation of expanded polyglutamine repeat androgen receptor in a cellular model of spinal and bulbar muscular atrophy. Human molecular genetics. 2002;11(5):515–23. Epub 2002/03/05. 11875046 10.1093/hmg/11.5.515

[9] Calamini B, Silva MC, Madoux F, Hutt DM, Khanna S, Chalfant MA, et al Small-molecule proteostasis regulators for protein conformational diseases. Nature chemical biology. 2012;8(2):185–96. Epub 2011/12/27. PubMed Central PMCID: PMC3262058. 10.1038/nchembio.763 PMC3262058 22198733

[10] Chai Y, Koppenhafer SL, Bonini NM, Paulson HL. Analysis of the role of heat shock protein (Hsp) molecular chaperones in polyglutamine disease. The Journal of neuroscience: the official journal of the Society for Neuroscience. 1999;19(23):10338–47. Epub 1999/11/27. 10575031 10.1523/JNEUROSCI.19-23-10338.1999 PMC6782415

[11] Fujikake N, Nagai Y, Popiel HA, Okamoto Y, Yamaguchi M, Toda T. Heat shock transcription factor 1-activating compounds suppress polyglutamine-induced neurodegeneration through induction of multiple molecular chaperones. The Journal of biological chemistry. 2008;283(38):26188–97. Epub 2008/07/18. 10.1074/jbc.M710521200 18632670 PMC3258858

[12] Prahlad V, Morimoto RI. Neuronal circuitry regulates the response of Caenorhabditis elegans to misfolded proteins. Proceedings of the National Academy of Sciences of the United States of America. 2011;108(34):14204–9. Epub 2011/08/17. PubMed Central PMCID: PMC3161566. 10.1073/pnas.1106557108 21844355 PMC3161566

[13] Sakahira H, Breuer P, Hayer-Hartl MK, Hartl FU. Molecular chaperones as modulators of polyglutamine protein aggregation and toxicity. Proceedings of the National Academy of Sciences of the United States of America. 2002;99 Suppl 4:16412–8. Epub 2002/08/22. PubMed Central PMCID: PMC139902. 12189209 10.1073/pnas.182426899 PMC139902

[14] Wacker JL, Zareie MH, Fong H, Sarikaya M, Muchowski PJ. Hsp70 and Hsp40 attenuate formation of spherical and annular polyglutamine oligomers by partitioning monomer. Nature structural & molecular biology. 2004;11(12):1215–22. Epub 2004/11/16. 10.1038/nsmb860 15543156

[15] Warrick JM, Chan HY, Gray-Board GL, Chai Y, Paulson HL, Bonini NM. Suppression of polyglutamine-mediated neurodegeneration in Drosophila by the molecular chaperone HSP70. Nature genetics. 1999;23(4):425–8. Epub 1999/12/02. 10.1038/70532 10581028

[16] Butterworth NJ, Williams L, Bullock JY, Love DR, Faull RL, Dragunow M. Trinucleotide (CAG) repeat length is positively correlated with the degree of DNA fragmentation in Huntington's disease striatum. Neuroscience. 1998;87(1):49–53. Epub 1998/08/29. 9722140 10.1016/s0306-4522(98)00129-8

[17] Mattson MP. Apoptosis in neurodegenerative disorders. Nature reviews Molecular cell biology. 2000;1(2):120–9. Epub 2001/03/20. 10.1038/35040009 11253364

[18] Goldberg YP, Nicholson DW, Rasper DM, Kalchman MA, Koide HB, Graham RK, et al Cleavage of huntingtin by apopain, a proapoptotic cysteine protease, is modulated by the polyglutamine tract. Nature genetics. 1996;13(4):442–9. Epub 1996/08/01. 10.1038/ng0896-442 8696339

[19] Wellington CL, Ellerby LM, Hackam AS, Margolis RL, Trifiro MA, Singaraja R, et al Caspase cleavage of gene products associated with triplet expansion disorders generates truncated fragments containing the polyglutamine tract. The Journal of biological chemistry. 1998;273(15):9158–67. Epub 1998/05/16. 9535906 10.1074/jbc.273.15.9158

[20] Wellington CL, Singaraja R, Ellerby L, Savill J, Roy S, Leavitt B, et al Inhibiting caspase cleavage of huntingtin reduces toxicity and aggregate formation in neuronal and nonneuronal cells. The Journal of biological chemistry. 2000;275(26):19831–8. Epub 2000/04/20. 10.1074/jbc.M001475200 10770929

[21] Landles C, Sathasivam K, Weiss A, Woodman B, Moffitt H, Finkbeiner S, et al Proteolysis of mutant huntingtin produces an exon 1 fragment that accumulates as an aggregated protein in neuronal nuclei in Huntington disease. The Journal of biological chemistry. 2010;285(12):8808–23. Epub 2010/01/21. PubMed Central PMCID: PMC2838303. 10.1074/jbc.M109.075028 20086007 PMC2838303

[22] Bates GP, Dorsey R, Gusella JF, Hayden MR, Kay C, Leavitt BR, et al Huntington disease. Nature reviews Disease primers. 2015;1:15005 Epub 2015/01/01. 10.1038/nrdp.2015.5 27188817

[23] Sahoo B, Singer D, Kodali R, Zuchner T, Wetzel R. Aggregation behavior of chemically synthesized, full-length huntingtin exon1. Biochemistry. 2014;53(24):3897–907. Epub 2014/06/13. PubMed Central PMCID: PMC4075985. 10.1021/bi500300c 24921664 PMC4075985

[24] Sahl SJ, Weiss LE, Duim WC, Frydman J, Moerner WE. Cellular inclusion bodies of mutant huntingtin exon 1 obscure small fibrillar aggregate species. Scientific reports. 2012;2:895 Epub 2012/11/30. PubMed Central PMCID: PMC3508451. 10.1038/srep00895 23193437 PMC3508451

[25] Mangiarini L, Sathasivam K, Seller M, Cozens B, Harper A, Hetherington C, et al Exon 1 of the HD gene with an expanded CAG repeat is sufficient to cause a progressive neurological phenotype in transgenic mice. Cell. 1996;87(3):493–506. Epub 1996/11/01. 8898202 10.1016/s0092-8674(00)81369-0

[26] Zhang X, Smith DL, Meriin AB, Engemann S, Russel DE, Roark M, et al A potent small molecule inhibits polyglutamine aggregation in Huntington's disease neurons and suppresses neurodegeneration in vivo. Proceedings of the National Academy of Sciences of the United States of America. 2005;102(3):892–7. Epub 2005/01/12. PubMed Central PMCID: PMC545525. 10.1073/pnas.0408936102 15642944 PMC545525

[27] Faber PW, Alter JR, MacDonald ME, Hart AC. Polyglutamine-mediated dysfunction and apoptotic death of a Caenorhabditis elegans sensory neuron. Proceedings of the National Academy of Sciences of the United States of America. 1999;96(1):179–84. 9874792 10.1073/pnas.96.1.179 PMC15113

[28] Parker JA, Connolly JB, Wellington C, Hayden M, Dausset J, Neri C. Expanded polyglutamines in Caenorhabditis elegans cause axonal abnormalities and severe dysfunction of PLM mechanosensory neurons without cell death. Proceedings of the National Academy of Sciences of the United States of America. 2001;98(23):13318–23. 10.1073/pnas.231476398 11687635 PMC60868

[29] Wang H, Lim PJ, Yin C, Rieckher M, Vogel BE, Monteiro MJ. Suppression of polyglutamine-induced toxicity in cell and animal models of Huntington's disease by ubiquilin. Human molecular genetics. 2006;15(6):1025–41. Epub 2006/02/08. 10.1093/hmg/ddl017 16461334

[30] Morley JF, Brignull HR, Weyers JJ, Morimoto RI. The threshold for polyglutamine-expansion protein aggregation and cellular toxicity is dynamic and influenced by aging in Caenorhabditis elegans. Proceedings of the National Academy of Sciences of the United States of America. 2002;99(16):10417–22. 10.1073/pnas.152161099 12122205 PMC124929

[31] Brignull HR, Moore FE, Tang SJ, Morimoto RI. Polyglutamine proteins at the pathogenic threshold display neuron-specific aggregation in a pan-neuronal Caenorhabditis elegans model. The Journal of neuroscience: the official journal of the Society for Neuroscience. 2006;26(29):7597–606. 16855087 10.1523/JNEUROSCI.0990-06.2006 PMC6674286

[32] Gidalevitz T, Ben-Zvi A, Ho KH, Brignull HR, Morimoto RI. Progressive disruption of cellular protein folding in models of polyglutamine diseases. Science. 2006;311(5766):1471–4. Epub 2006/02/14. 10.1126/science.1124514 16469881

[33] Gidalevitz T, Wang N, Deravaj T, Alexander-Floyd J, Morimoto RI. Natural genetic variation determines susceptibility to aggregation or toxicity in a C. elegans model for polyglutamine disease. BMC biology. 2013;11(1):100. Epub 2013/10/02. 24079614 10.1186/1741-7007-11-100 PMC3816611

[34] Nollen EA, Garcia SM, van Haaften G, Kim S, Chavez A, Morimoto RI, et al Genome-wide RNA interference screen identifies previously undescribed regulators of polyglutamine aggregation. Proceedings of the National Academy of Sciences of the United States of America. 2004;101(17):6403–8. 10.1073/pnas.0307697101 15084750 PMC404057

[35] Lejeune FX, Mesrob L, Parmentier F, Bicep C, Vazquez-Manrique RP, Parker JA, et al Large-scale functional RNAi screen in C. elegans identifies genes that regulate the dysfunction of mutant polyglutamine neurons. BMC genomics. 2012;13:91 Epub 2012/03/15. PubMed Central PMCID: PMC3331833. 10.1186/1471-2164-13-91 22413862 PMC3331833

[36] Parker JA, Vazquez-Manrique RP, Tourette C, Farina F, Offner N, Mukhopadhyay A, et al Integration of beta-catenin, sirtuin, and FOXO signaling protects from mutant huntingtin toxicity. The Journal of neuroscience: the official journal of the Society for Neuroscience. 2012;32(36):12630–40. Epub 2012/09/08. PubMed Central PMCID: PMC3780431. 22956852 10.1523/JNEUROSCI.0277-12.2012 PMC3780431

[37] Tourette C, Farina F, Vazquez-Manrique RP, Orfila AM, Voisin J, Hernandez S, et al The Wnt receptor Ryk reduces neuronal and cell survival capacity by repressing FOXO activity during the early phases of mutant huntingtin pathogenicity. PLoS biology. 2014;12(6):e1001895 Epub 2014/06/25. PubMed Central PMCID: PMC4068980. 10.1371/journal.pbio.1001895 24960609 PMC4068980

[38] Zielonka D, Piotrowska I, Marcinkowski JT, Mielcarek M. Skeletal muscle pathology in Huntington's disease. Frontiers in physiology. 2014;5:380 Epub 2014/10/24. PubMed Central PMCID: PMC4186279. 10.3389/fphys.2014.00380 25339908 PMC4186279

[39] Reddy PH. Increased mitochondrial fission and neuronal dysfunction in Huntington's disease: implications for molecular inhibitors of excessive mitochondrial fission. Drug discovery today. 2014;19(7):951–5. Epub 2014/04/01. PubMed Central PMCID: PMC4191657. 10.1016/j.drudis.2014.03.020 24681059 PMC4191657

[40] Lodi R, Schapira AH, Manners D, Styles P, Wood NW, Taylor DJ, et al Abnormal in vivo skeletal muscle energy metabolism in Huntington's disease and dentatorubropallidoluysian atrophy. Annals of neurology. 2000;48(1):72–6. Epub 2000/07/14. 10894218

[41] Wang H, Lim PJ, Karbowski M, Monteiro MJ. Effects of overexpression of huntingtin proteins on mitochondrial integrity. Human molecular genetics. 2009;18(4):737–52. Epub 2008/11/29. PubMed Central PMCID: PMC2722218. 10.1093/hmg/ddn404 19039036 PMC2722218

[42] Andrade MA, Bork P. HEAT repeats in the Huntington's disease protein. Nature genetics. 1995;11(2):115–6. Epub 1995/10/01. 10.1038/ng1095-115 7550332

[43] Hodgson JG, Agopyan N, Gutekunst CA, Leavitt BR, LePiane F, Singaraja R, et al A YAC mouse model for Huntington's disease with full-length mutant huntingtin, cytoplasmic toxicity, and selective striatal neurodegeneration. Neuron. 1999;23(1):181–92. 10402204 10.1016/s0896-6273(00)80764-3

[44] Saudou F, Finkbeiner S, Devys D, Greenberg ME. Huntingtin acts in the nucleus to induce apoptosis but death does not correlate with the formation of intranuclear inclusions. Cell. 1998;95(1):55–66. Epub 1998/10/20. 9778247 10.1016/s0092-8674(00)81782-1

[45] Brenner S. The genetics of Caenorhabditis elegans. Genetics. 1974;77(1):71–94. Epub 1974/05/01. PubMed Central PMCID: PMC1213120. 4366476 10.1093/genetics/77.1.71 PMC1213120

[46] Kenyon C, Chang J, Gensch E, Rudner A, Tabtiang R. A C. elegans mutant that lives twice as long as wild type. Nature. 1993;366(6454):461–4. 10.1038/366461a0 8247153

[47] Duyao M, Ambrose C, Myers R, Novelletto A, Persichetti F, Frontali M, et al Trinucleotide repeat length instability and age of onset in Huntington's disease. Nature genetics. 1993;4(4):387–92. Epub 1993/08/01. 10.1038/ng0893-387 8401587

[48] Snell RG, MacMillan JC, Cheadle JP, Fenton I, Lazarou LP, Davies P, et al Relationship between trinucleotide repeat expansion and phenotypic variation in Huntington's disease. Nature genetics. 1993;4(4):393–7. Epub 1993/08/01. 10.1038/ng0893-393 8401588

[49] Andrew SE, Goldberg YP, Kremer B, Telenius H, Theilmann J, Adam S, et al The relationship between trinucleotide (CAG) repeat length and clinical features of Huntington's disease. Nature genetics. 1993;4(4):398–403. Epub 1993/08/01. 10.1038/ng0893-398 8401589

[50] Bhat KP, Yan S, Wang CE, Li S, Li XJ. Differential ubiquitination and degradation of huntingtin fragments modulated by ubiquitin-protein ligase E3A. Proceedings of the National Academy of Sciences of the United States of America. 2014;111(15):5706–11. Epub 2014/04/08. PubMed Central PMCID: PMC3992696. 10.1073/pnas.1402215111 24706802 PMC3992696

[51] Christie NT, Lee AL, Fay HG, Gray AA, Kikis EA. Novel polyglutamine model uncouples proteotoxicity from aging. PloS one. 2014;9(5):e96835 Epub 2014/05/13. PubMed Central PMCID: PMC4016013. 10.1371/journal.pone.0096835 24817148 PMC4016013

