# Supplementary figures and images for: A new Caenorhabditis elegans model of human huntingtin 513 aggregation and toxicity in body wall muscles
Source: PLoS One. 2017 Mar 10;12(3):e0173644. doi: 10.1371/journal.pone.0173644 (PMC5345860; doi:10.1371/journal.pone.0173644)

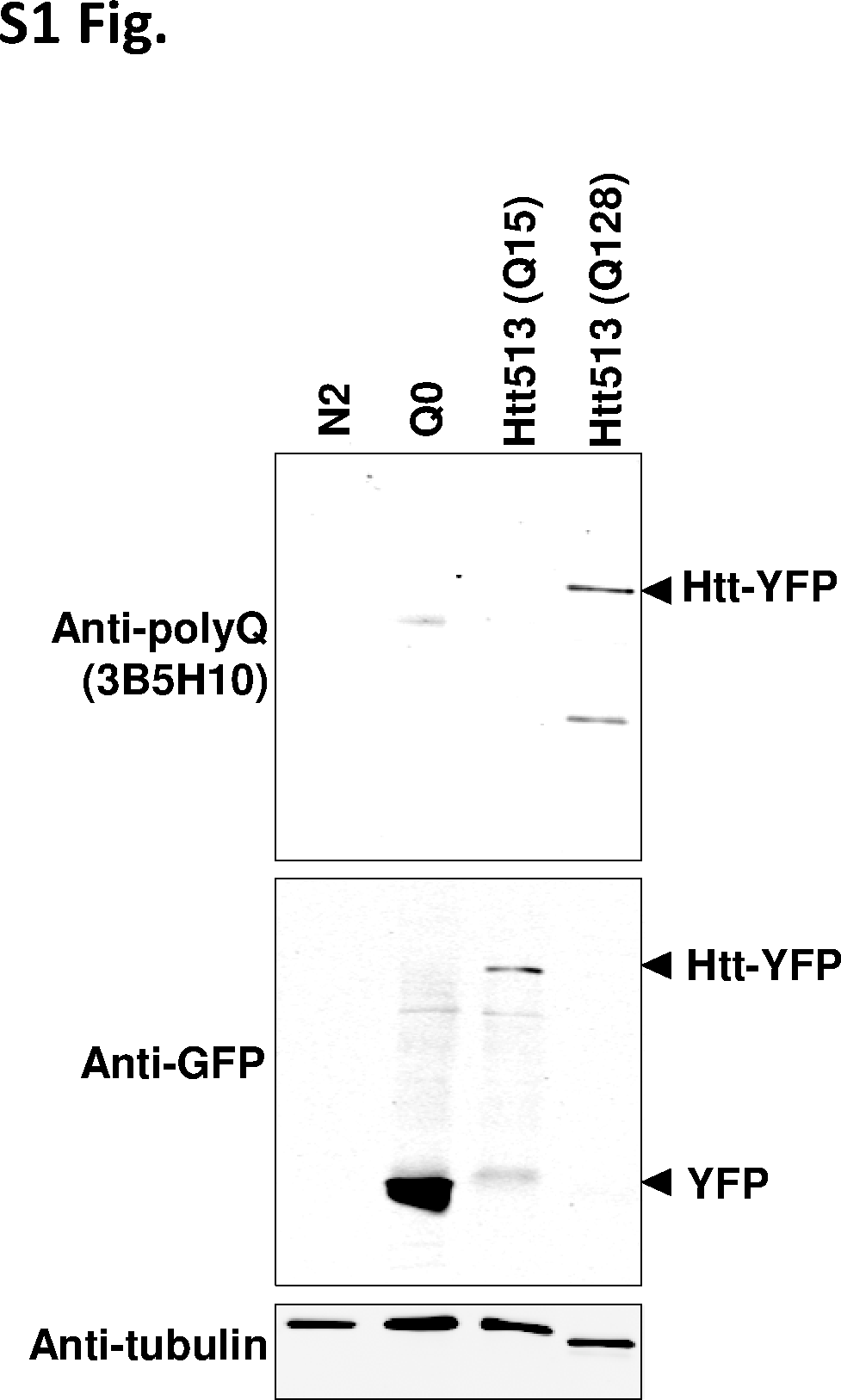

Supplement: S1 Fig — Top: Representative immunoblot probed with an anti-expanded polyQ antibody. Bottom: The same immunoblot as above probed with an anti-GFP antibody. The YFP control was expressed from an integrated transgene while Htt513(Q15) and Htt513(Q128) were expressed from extrachromosomal arrays. (TIF) [file pone.0173644.s001.tif]

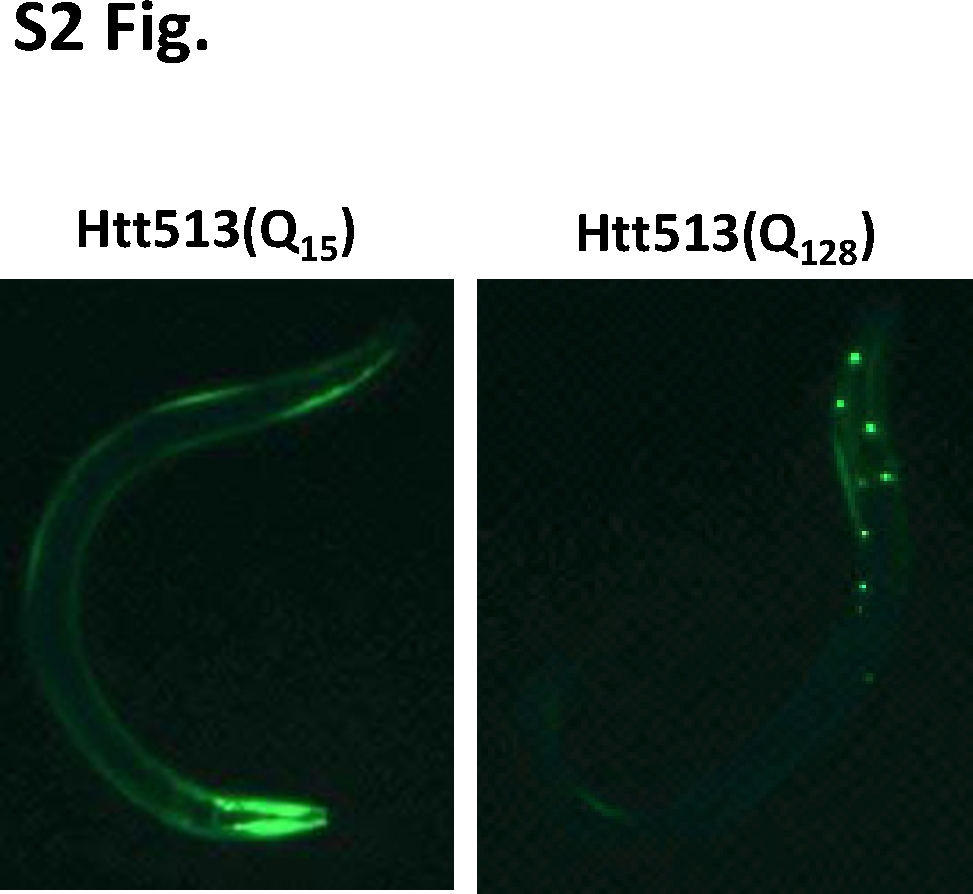

Supplement: S2 Fig — Day 1 adult animals were fixed and imaged with a compound fluorescence microscope. YFP fluorescence is shown for animals expressing Htt513(Q15) or Htt513(Q128). (TIF) [file pone.0173644.s002.tif]

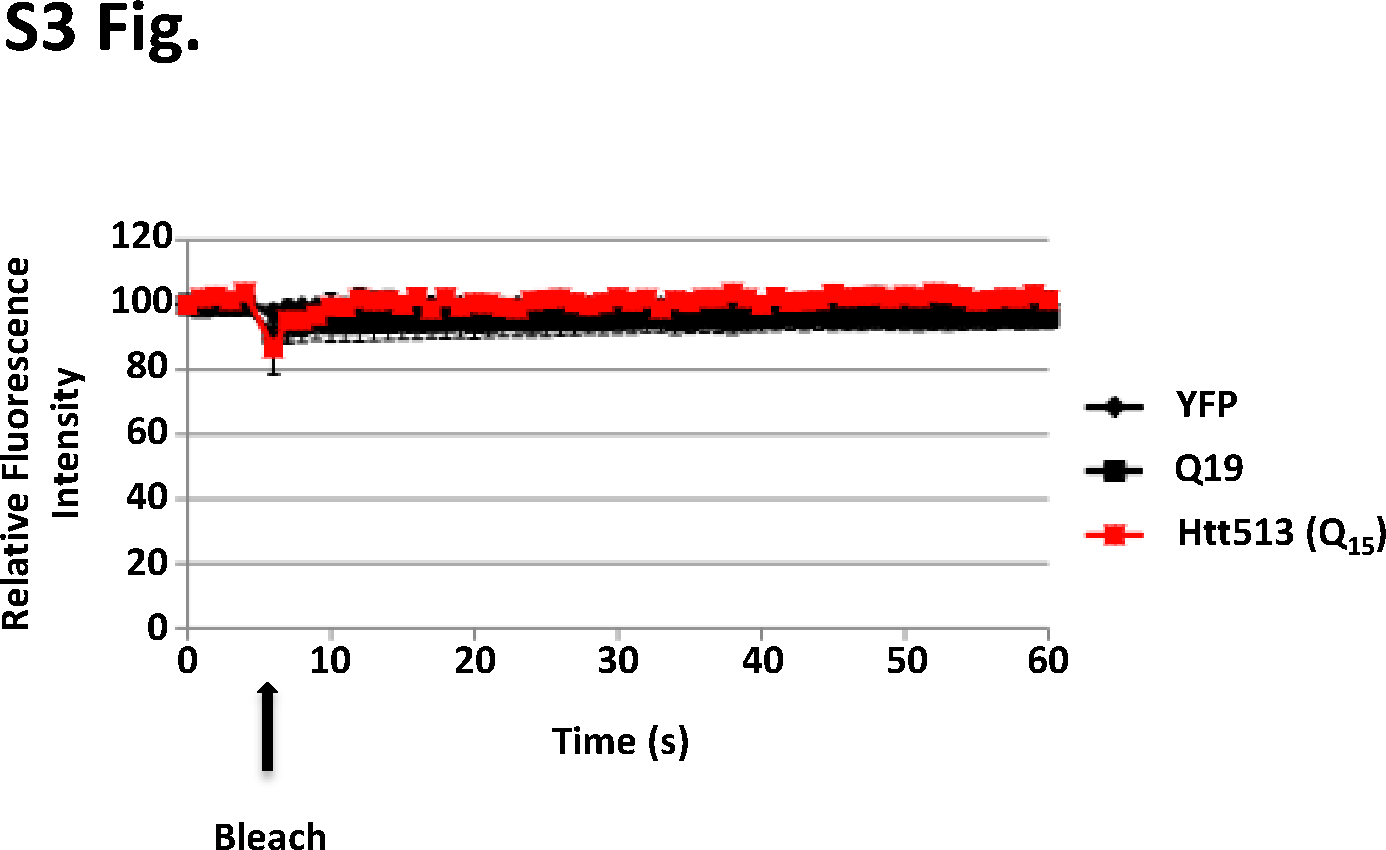

Supplement: S3 Fig — FRAP was performed on regions of diffuse fluorescence in animals expressing YFP alone, Q19-YFP or Htt513(Q15). Quantification of relative fluorescence intensity over a 60s FRAP time course is shown. Data represent averages of at least 10 regions of diffuse fluorescence in different animals. Error bars represent standard error of the mean. The time of bleaching is indicated with an arrow. (TIF) [file pone.0173644.s003.tif]
